# GABA promotes survival and axonal regeneration in identifiable descending neurons after spinal cord injury in larval lampreys

**DOI:** 10.1038/s41419-018-0704-9

**Published:** 2018-06-28

**Authors:** Daniel Romaus-Sanjurjo, Rocío Ledo-García, Blanca Fernández-López, Kendra Hanslik, Jennifer R. Morgan, Antón Barreiro-Iglesias, María Celina Rodicio

**Affiliations:** 1 Department of Functional Biology, CIBUS, Faculty of Biology, Universidade de Santiago de Compostela, 15782 Santiago de Compostela, Spain; 2 Eugene Bell Center for Regenerative Biology and Tissue Engineering; Marine Biological Laboratory, 7 MBL St., Woods Hole, MA 02543 USA; 3 Present Address: Department of Anatomy, Neuroscience Center, University of Helsinki, Haartmaninkatu 8, 00014 Helsinki, Finland

## Abstract

The poor regenerative capacity of descending neurons is one of the main causes of the lack of recovery after spinal cord injury (SCI). Thus, it is of crucial importance to find ways to promote axonal regeneration. In addition, the prevention of retrograde degeneration leading to the atrophy/death of descending neurons is an obvious prerequisite to activate axonal regeneration. Lampreys show an amazing regenerative capacity after SCI. Recent histological work in lampreys suggested that GABA, which is massively released after a SCI, could promote the survival of descending neurons. Here, we aimed to study if GABA, acting through GABAB receptors, promotes the survival and axonal regeneration of descending neurons of larval sea lampreys after a complete SCI. First, we used in situ hybridization to confirm that identifiable descending neurons of late-stage larvae express the gabab1 subunit of the GABAB receptor. We also observed an acute increase in the expression of this subunit in descending neurons after SCI, which further supported the possible role of GABA and GABAB receptors in promoting the survival and regeneration of these neurons. So, we performed gain and loss of function experiments to confirm this hypothesis. Treatments with GABA and baclofen (GABAB agonist) significantly reduced caspase activation in descending neurons 2 weeks after a complete SCI. Long-term treatments with GABOB (a GABA analogue) and baclofen significantly promoted axonal regeneration of descending neurons after SCI. These data indicate that GABAergic signalling through GABAB receptors promotes the survival and regeneration of descending neurons after SCI. Finally, we used morpholinos against the gabab1 subunit to knockdown the expression of the GABAB receptor in descending neurons. Long-term morpholino treatments caused a significant inhibition of axonal regeneration. This shows that endogenous GABA promotes axonal regeneration after a complete SCI in lampreys by activating GABAB receptors.

## Introduction

In contrast to mammals, lampreys show spontaneous and successful functional recovery after a complete spinal cord injury (SCI) and this is in part due to their impressive ability for axonal regeneration^1–8^. But, even in lampreys, not all descending neurons of the brain are able to regenerate their axons through the site of injury after a complete spinal cord transection^4,9–12^. The lamprey brainstem contains approximately 30 large individually identifiable descending reticulospinal neurons that vary greatly in their ability for axonal regeneration after SCI, even when their axons run in similar paths in a spinal cord that is permissive for axonal regrowth^4,12,13^. Some identifiable descending neurons of lampreys are considered “good regenerators” (i.e. they regenerate their axon more than 55% of the times; the I3, I4, I5, B2, B5 and B6 neurons) and others are considered “bad regenerators” (i.e. they regenerate their axon less than 50% of the times; the M1, M2, M3, I1, I2, B1, B3, B4 and Mth neurons)^4,6,12^. This indicates that interactions with the extrinsic spinal cord environment and intrinsic differences between descending neurons affect their regenerative abilities after SCI. Recent work has also shown that identifiable descending neurons of lampreys that are known to be “bad regenerators” slowly die after a complete SCI and are also “poor survivors”^12,14,15^. The death of these neurons after SCI appears to be apoptotic as indicated by the appearance of TUNEL labelling and activated caspases in their soma^14–18^. This offers a convenient vertebrate model to study the inhibition or promotion of neuronal survival and axonal regeneration in the same in vivo preparation and at the level of single neurons.

In mammals, SCI leads to a massive release of aminoacidergic neurotransmitters (glycine and GABA:^19,20^; glutamate:^21–23^). Excessive glutamate release after SCI is responsible for excitotoxicity and neuronal death^21,22^. High extracellular glutamate levels result in excessive activation of glutamate receptors, triggering massive Ca^2+^ influx into cells, which leads to neuronal death^24^. Extracellular glycine could also contribute to glutamate excitotoxicity^20^, since it is a co-agonist of the *N*-methyl-D-aspartate glutamate receptor^25^. The phenomenon of excitotoxicity has been mainly studied in intrinsic spinal cord cells; however, retrograde damage to neurons is also likely due to the fact that Ca^2+^ ions gain access to the axoplasm of damaged axons^26^. In contrast to glutamate, it has been reported that GABA could have neuroprotective effects after different types of central nervous system (CNS) damage^27–31^. The activation of pre-synaptic GABAB receptors causes inactivation of voltage-dependent Ca^2+^ channels (see^32^), which could prevent the influx of Ca^2+^ ions due to glutamate release. In addition, it has been shown that GABA can modulate and promote neurite outgrowth in vitro or during development (for reviews see^33,34^). However, a role for GABA and GABAB receptors in neuroprotection and especially in axonal regeneration after SCI has not been reported yet.

In lampreys, glutamate induces an inhibition of neurite outgrowth in reticulospinal neurons in vitro due to Ca^2+^ influx^35^. Electrophysiological studies have also suggested that low intracellular Ca^2+^ levels due to downregulation of Ca^2+^ channels could facilitate axonal regeneration in axotomized descending neurons of lampreys^36^. More recently, we have reported that, as in mammals, there is a massive release of glutamate, GABA and glycine from most spinal cord neurons close to the lesion site following a complete SCI^37–39^. Between 1 and 3 days after the injury, we observed the extracellular accumulation of GABA in the form of “*halos*” around some axotomized axons of descending neurons close to the site of injury. Statistical analyses revealed a significant correlation between GABA accumulation and a higher survival ability of the corresponding identifiable descending neurons^37^. An electrophysiological study in the spinal cord of lampreys has also found a correlation between higher GABAergic inhibition and a better recovery of function in spinal lesioned animals^40^. These data prompted us to hypothesize that, in lampreys, increased GABA signalling after SCI could be favouring the recovery process by promoting survival and axonal regeneration of descending neurons. Here, we address this question for the first time in vivo in any vertebrate and provide gain and loss of function evidence showing that endogenous GABA, acting through GABAB receptors, promotes survival and axonal regeneration of identifiable descending neurons after SCI in lampreys.

## Materials and methods

### Animals

All experiments involving animals were approved by the Bioethics Committee at the University of Santiago de Compostela and the *Consellería do Medio Rural e do Mar* of the *Xunta de Galicia* (License reference JLPV/IId; Galicia, Spain) or the Institutional Animal Care and Use Committee at the Marine Biological Laboratory (Woods Hole, MA) and were performed in accordance to European Union and Spanish guidelines on animal care and experimentation or the National Institutes of Health, respectively. During experimental procedures, special effort was taken to minimize animal suffering and to reduce the use of animals. Animals were deeply anaesthetized with 0.1% MS-222 (Sigma, St. Louis, MO) in lamprey Ringer solution before all experimental procedures and euthanized by decapitation at the end of the experiments.

Mature and developmentally stable larval sea lampreys, *Petromyzon marinus* L. (*n* = 115; between 95 and 120 mm in body length, 5 to 7 years of age), were used in the study. Larval lampreys were collected from the river Ulla (Galicia, Spain), with permission from the *Xunta de Galicia*, or provided by Lamprey Services, Inc. (Ludington, MI, USA) and maintained in aerated fresh water aquaria at 15–23 °C with a bed of river sediment until their use in experimental procedures. Lampreys were randomly distributed between the different experimental groups.

### SCI surgical procedures

Animals were assigned to the following experimental groups: control unlesioned animals or animals with a complete spinal cord transection that were analyzed 1 week post-lesion (wpl), 2 wpl, 4 wpl, 10 wpl or 12 wpl. Within the 2, 10 and 12 wpl groups, the injured animals were assigned to either control or treatment groups. Table 1 summarizes the number of animals assigned to each experimental group and condition. Each experiment was carried out in at least two different batches of animals. Complete spinal cord transections were performed as previously described^41^. Briefly, the rostral spinal cord was exposed from the dorsal midline at the level of the 5th gill by making a longitudinal incision with a scalpel (#11). A complete spinal cord transection was performed with Castroviejo scissors and the spinal cord cut ends were visualized under the stereomicroscope. After spinal transections, the animals were returned to fresh water tanks and each transected animal was examined 24 h after surgery to confirm that there was no movement caudal to the lesion site. Then, the animals were allowed to recover in individual fresh water tanks at 19.5 °C and in the dark.

**Table 1:** Table showing the number of animals included in each experimental group and also the total number of identifiable descending neurons that were included in the analyses

		Animals	Total number of neurons included in the analyses														
			M1	M2	M3	I1	I2	I3	I4	I5	B1	B2	B3	B4	B5	B6	Mth
Changes in gabab1 expression	Control	7	11	11	12	8	–	5	8	5	9	–	10	9	–	7	9
	1 wpl	7	10	10	14	12	–	12	9	6	9	–	13	9	–	11	14
	4 wpl	6	10	12	12	9	–	6	6	6	5	–	6	5	–	5	7
GABA treatment (2 wpl)	Control	6*	11	11	10	12	9	11	12	7	12	11	12	12	11	12	12
	Treated	5	6	8	8	9	6	9	9	5	10	10	10	10	7	9	10
Baclofen treatment (caspase activation, 2 wpl)	Control	7*	13	12	11	14	9	13	14	8	14	13	14	14	12	14	14
	Treated	7	12	13	13	14	7	14	13	6	14	13	14	14	13	13	13
GABOB treatment (12 wpl)	Control	15	30	30	29	29	24	30	30	29	30	30	30	30	30	30	30
	Treated	14	26	25	24	28	24	28	27	28	25	24	26	28	23	28	24
Baclofen treatment (axonal regeneration, 12 wpl)	Control	7	12	12	13	14	12	14	14	11	14	12	14	14	14	12	13
	Treated	11	21	21	21	22	19	22	22	21	22	22	22	22	22	22	22
Gabab1 morpholino (ISH, 2 wpl)	Control	3	6	–	–	6	–	–	–	–	–	–	–	–	–	–	–
	Treated	4	7	–	–	8	–	–	–	–	–	–	–	–	–	–	–
Gabab1 morpholino (axonal regeneration, 10 wpl)	Control	9	18	18	18	18	18	18	18	18	18	18	18	18	18	18	18
	Treated	13	26	26	26	25	26	26	26	26	26	26	26	26	26	26	26
	Total	115															

Please note that in the in situ hybridization experiments, only the neurons that were unequivocally identified in at least two brain sections were included in the quantifications. In the FLICA experiments, six animals were used as controls for both the GABA and baclofen treatments and an extra animal was used as a control only for the baclofen treatment (asterisks)

### In situ hybridization

For gabab1 in situ hybridization, the head of the animals was fixed by immersion in 4% paraformaldehyde (PFA) in 0.05 M Tris-buffered saline (TBS; pH 7.4) for 12 h. Then, the brains were dissected out, washed and embedded in Neg 50^TM^ (Microm International GmbH, Walldorf, Germany), frozen in liquid nitrogen-cooled isopentane, sectioned on a cryostat in the transverse plane (14 μm thick) and mounted on Superfrost Plus glass slides (Menzel, Braunschweig, Germany). In situ hybridization with a specific riboprobe for the gabab1 subunit of the sea lamprey gabab receptor (GenBank accession number KX655780; see Suppl. Figure 1) was conducted as previously described^42^. Briefly, brain sections were incubated with the sea lamprey gabab1 DIG-labelled probe at 70 °C and treated with RNAse A (Invitrogen, Massachusetts, USA) in the post-hybridization washes. Then, the sections were incubated with a sheep anti-DIG antibody conjugated to alkaline phosphatase (1:2000; Roche, Mannhein, Germany) overnight. Staining was conducted in BM Purple (Roche) at 37 °C. In situ hybridization experiments were performed in parallel with animals from the different experimental groups (control, 1 wpl, 2 wpl and 4 wpl) and the colorimetric reaction was stopped simultaneously for all sections from the different groups of animals.

### Drug treatments

The following drugs were used to treat the animals following the complete spinal cord transection: GABA (Sigma; Cat#: A2129; MW: 103.12 g/mol), GABOB (a GABA analogue; Sigma; Cat#: A56655; MW: 119.12 g/mol) and baclofen (a GABAB receptor agonist). Baclofen was acquired from two different companies: for the experiments of caspase activation, we used baclofen from Molekula (Newcastle, UK; Cat#: 31184509; MW: 213.66 g/mol), and for the experiments of axonal regeneration, we used baclofen from Carbosynth (Berkshire, UK; Cat#: FB18127; MW: 213.66 g/mol). The drugs were applied in the water where the animals were left after the SCI surgical procedures (GABA at a concentration of 500 µM, GABOB at a concentration of 50 µM and baclofen at a concentration of 125 µM). The concentrations of baclofen and GABA were selected based on previous in vitro electrophysiological studies in lampreys^43^. Since GABA does not easily cross the blood–brain barrier, it was applied at a high concentration and only in the first days after the injury when the spinal cord is still disrupted. We assumed that GABOB and baclofen also cross the blood–brain barrier as in mammals, since the blood–brain barrier of lampreys is similar to that of higher vertebrates^44,45^. While we do not know the final concentration of the drugs in the CNS, we were confident that this application route allows access to the CNS as there were changes in the swimming behaviour of unlesioned animals in pilot experiments using baclofen and GABOB at these concentrations (not shown). Since these drugs are water soluble, control lesioned and non-treated animals were left in fresh water only. The animals that were analyzed for caspase activation 2 wpl were treated with GABA or baclofen during 4 days from the day of injury and replacing the drug and water every day during those 4 days. The animals that were analyzed for axonal regeneration 12 wpl were treated with GABOB or baclofen during the 12 weeks replacing the drug and the water four times each week. The animals were always kept in the dark during the drug treatments to prevent light degradation of these drugs.

### Morpholino treatment

Application of morpholinos was performed as previously described in ref.^13^. Briefly, the spinal cord was transected at the level of the 5th gill (see surgical procedures), and morpholinos (20 µg in lamprey internal solution: 180 mM KCl, 10 mM HEPES, pH 7.4; designed by GeneTools, LLC; Philomath, OR) were added at the time and site of SCI soaked in a small piece of Gelfoam (Pfizer; New York, NY). These included an active splicing-blocking gabab1 morpholino (5′-ACGTCTGCAACGGAGAGTCATGAGA-3′) generated against the boundary between the second intron and the second exon of the partial sea lamprey gabab1 sequence (Suppl. Figure 1), and a 5-base pair mismatch gabab1 negative control morpholino (5′-ACcTCTcCAACcGAGAcTCATcAGA-3′). During recovery, the morpholinos are retrogradely transported to the cell soma of descending neurons where they can knockdown the expression of the target mRNA^13,46–48^. Animals were allowed to recover for 10 wpl to analyze the effect of gabab1 knockdown (KD) in axonal regeneration of identifiable descending neurons. In situ hybridization was used to control the efficacy of the gabab1 morpholino KD in animals processed at 2 wpl.

### Detection of activated caspases in whole-mounted brain preparations

The Image-iT LIVE Green Poly Caspases Detection Kit (Cat. No. I35104, Invitrogen, USA) was used to detect activated caspases in identifiable descending neurons (the M1, M2, M3, I1, I2, I3, I4, I5, B1, B2, B3, B4, B5, B6 and Mth neurons; see Suppl. Figure 2A) of larval sea lampreys 2 weeks after the complete spinal cord transection and the GABA or baclofen treatments. This kit contains 1 vial (component A of the kit) of the lyophilized FLICA reagent (FAM–VAD–FMK). The reagent associates a fluoromethyl ketone (FMK) moiety, which can react covalently with a cysteine, with a caspase-specific aminoacid sequence (valine–alanine–aspartic acid (VAD)). A carboxyfluorescein group (FAM) is attached as a fluorescent reporter. The FLICA reagent interacts with the enzyme active centre of an activated caspase via the recognition sequence, and then attaches covalently through the FMK moiety. Experiments for the detection of activated caspases in whole-mounted brain preparations were done as previously described^16–18^. Briefly, brains from control and treated 2 wpl animals were dissected out and immediately incubated in 150 µL of phosphate buffered saline (PBS) containing 1 μL of the 150× FLICA labelling solution at 37 °C for 1 h. Then, the brains were washed with PBS. Brains were fixed in 4% PFA in PBS for 2 h and 30 min at 4 °C. Next, the brains were washed with PBS, mounted on Superfrost Plus glass slides, and mounted with Mowiol.

### Retrograde labelling of descending neurons with regenerated axons

At 10 (morpholino treatments) or 12 (GABOB and baclofen treatments) wpl, a second complete spinal cord transection was performed 5 mm below the site of the original transection and the retrograde tracer Neurobiotin (NB, 322.8 Da molecular weight; Vector; Burlingame, CA) was applied to the spinal cord lesion with the aid of a Minutien pin (#000). The animals were allowed to recover at 19.5 °C with appropriate ventilation conditions for 7 days to allow the transport of the tracer from the application point to the neuronal soma of identifiable descending neurons (the M1, M2, M3, I1, I2, I3, I4, I5, B1, B2, B3, B4, B5, B6 and Mth were analyzed; see Suppl. Figure 2A). Since the original SCI also was a complete spinal cord transection, only neurons whose axons regenerated at least 5 mm below the site of injury were labelled by the tracer. Brains of these larvae were dissected out, and the posterior and cerebrotectal commissures of the brain were cut along the dorsal midline, and the alar plates were deflected laterally and pinned flat to a small strip of Sylgard (Dow Corning Co., USA) and fixed with 4% PFA in TBS for 4 h at room temperature. After washes in TBS, the brains were incubated at room temperature with Avidin D-FITC conjugated (Vector; Cat#: A-2001; 1:500) diluted in TBS containing 0.3% Triton X-100 for 2 days to reveal the presence of Neurobiotin. Brains were rinsed in TBS and distilled water and mounted with Mowiol.

### Imaging and quantifications

An Olympus photomicroscope (AX-70; Provis) with a 20× Apochromatic 0.75 lens and equipped with a colour digital camera (Olympus DP70, Tokyo, Japan) was used to acquire images of brain sections from the in situ hybridization experiments. Images were always acquired with the same microscope and software settings. For the quantification of the level of gabab1 positive signal in identifiable descending neurons, first we established the intensity rank of positive colorimetric in situ signal. For this, we analyzed 10 random images from different descending neurons of control and lesioned animals. The “histogram” function in Image J shows the number of pixels in each image in a range of intensity from 0 to 255. With these images, we compared the intensity values in regions with clear in situ signal and the intensity values in regions without in situ signal. Based on this, we established a value of 179 as the lower limit to consider the colorimetric in situ signal as positive. Then the number of pixels of positive in situ signal was quantified for each section of each identified descending neuron. In brain sections, the identification of some of the specific descending cells becomes more difficult than in whole-mounts. Thus, only the cells that were unequivocally identified in at least two different sections were included in the quantifications (the M1, M2, M3, I1, I3, I4, I5, B1, B3, B4, B6 and Mth neurons; see Suppl. Figure 2A). Then, we calculated the average number of positive pixels per section for each individual neuron (see Table 1) and these data were used for statistical analyses. The experimenter was blinded during quantifications.

The quantification of the intensity of FLICA labelling was done as previously described^18^. Briefly, photomicrographs were acquired with a spectral confocal microscope (model TCS-SP2; Leica, Wetzlar, Germany). Images were always acquired under the same microscope conditions for control or treated animals. Quantification of mean fluorescent intensity (mean grey value) of each identifiable neuron was done using the Fiji software. In whole-mounted brain preparations, the specific descending neurons are easily identifiable based on their morphology and rostro-caudal and dorso-ventral anatomical location. The experimenter was blinded during quantifications. The data from each individual identifiable neuron (see Table 1) were used for statistical analyses.

The percentage of neurons with regenerated axons (labelled by the Neurobiotin tracer) with respect to the total number of analyzed neurons (see Table 1) was calculated for each type of identifiable descending neuron using an Olympus microscope or a Zeiss AxioImager Z2 microscope. The percentage of neurons with regenerated axons with respect to the total number of analyzed neurons in each animal was also calculated and these data were used for statistical analyses. The experimenter was blinded during quantifications. For the figures, images were taken with the Olympus microscope or the spectral confocal microscope (model TCS-SP2; Leica).

After quantifications, contrast and brightness were minimally adjusted with Adobe Photoshop CS4 or CS6 (Adobe Systems, San José, CA, USA). Figure plates and lettering were generated using Adobe Photoshop CS4 or CS6 (Adobe Systems).

### Statistical analyses

Statistical analysis was carried out using Prism 6 (GraphPad software, La Jolla, CA). Data were presented as mean ± S.E.M. Normality of the data was determined only when *n* numbers were higher than 10 by using the D’Agostino-Pearson omnibus test, and the homoscedasticity was determined by the Brown-Forsythe test. The in situ hybridization data that were normally distributed and homoscedastic were analyzed by a one-way ANOVA. Post-hoc Dunnett’s multiple comparison tests were used to compare pairs of data. In situ hybridization data that were not normally distributed (or when the *n* numbers were lower than 10) were analyzed by a Kruskal–Wallis test and post-hoc Dunn’s multiple comparisons test. The results of control vs. treatment groups were analyzed by a Student’s *t*-test (for normally distributed data) or a Mann–Whitney *U* test (for non-normally distributed data). The in situ hybridization data after morpholino application were analyzed by a Mann–Whitney *U* test. The significance level was set at 0.05. In the figures, significance values were represented by different number of asterisks: 1 asterisk (*p* value between 0.01 and 0.05), 2 asterisks (*p* value between 0.001 and 0.01), 3 asterisks (*p* value between 0.0001 and 0.001) and 4 asterisks (*p* value <0.0001).

## Results

### Increased expression of the gabab1 subunit in identifiable descending neurons after SCI

**Fig. 1:**
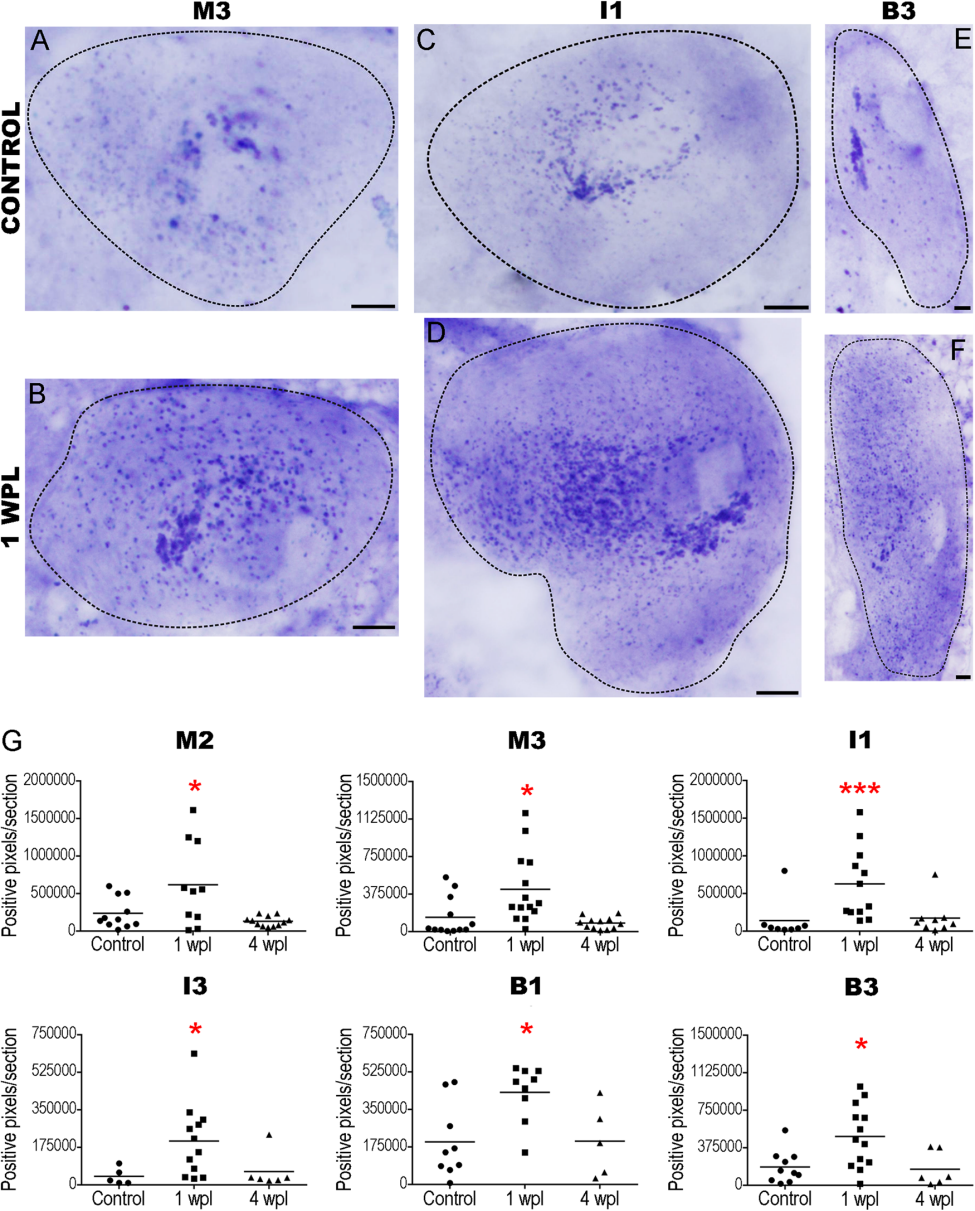
Changes in the expression of the gabab1 subunit in identifiable descending neurons after a complete SCI. **a**, **c** and **e** Photomicrographs of transverse sections of the brain showing the expression of the gabab1 transcript in descending neurons of control animals. **b**, **d** and **f** Photomicrographs of transverse sections of the brain showing the expression of the gabab1 transcript in descending neurons of lesioned animals at 1 wpl. **g** Graphs showing significant changes (asterisks) in the number of gabab1 positive pixels per section of the soma of identifiable descending neurons. The mean ± S.E.M. values are provided in Table 2. Scale bars: 20 µm

GABAB receptors are obligate heterodimers formed by gabab1 and gabab2 subunits^49^. In previous work, we reported the expression of the gabab1 and gabab2 receptor subunits in identifiable descending neurons of adult sea lampreys under normal conditions^42^. Here, we used gabab1 in situ hybridization first to confirm that this receptor is also expressed in identifiable descending neurons of mature larval sea lampreys (Suppl. Figure 2B; Fig. 1a, c, e) and then to quantify changes in its expression after SCI (Fig. 1a–g; Suppl. Figure 3). The M1, M2, M3, I1, I3, I4, I5, B1, B3, B4, B6 and Mth neurons were included in the analyses (see Material and Methods). This revealed a significant increase in the expression of the gabab1 subunit in the M2 (ANOVA, *p* = 0.0049), M3 (ANOVA, *p* = 0.002), I1 (Kruskal–Wallis, *p* = 0.0009), I3 (Kruskal–Wallis, *p* = 0.0097), B1 (Kruskal–Wallis, *p* = 0.015) and B3 (Kruskal–Wallis, *p* = 0.0178) neurons (Fig. 1g; Table 2) in 1 wpl animals as compared to control unlesioned animals. Subsequent power calculations (using 80% power) indicated that the sample sizes were appropriately powered. Although a similar trend was observed for the M1, I4, I5, B4, B6 and Mth neurons in 1 wpl animals as compared to control unlesioned animals, statistical analyses did not reveal significant changes in the expression of the gabab1 subunit in these neurons (Suppl. Figure 3; Table 2). At 4 wpl, the expression of the gabab1 subunit was not significantly different to control unlesioned animals in all identifiable descending neurons and returned to control levels (Fig. 1g; Suppl. Figure 3; Table 2). This shows that the complete SCI induced an acute increase in the expression of the gabab1 subunit in descending neurons, which, together with the accumulation of GABA around the axons of identifiable neurons^37^, supports the possible role of endogenous GABA as a neuroprotective and pro-regenerative molecule after SCI in lampreys.

**Table 2:** Mean ± S.E.M. values of the number of gabab1 positive in situ pixels/section in identifiable descending neurons of control and injured animals

Gabab1 positive pixels/section	Control	1 wpl	4 wpl
M1	258,170 ± 86,006	320,350 ± 78,104	148,657 ± 43,920
M2	237,552 ± 61,602	618,158 ± 176,397	131,367 ± 19,089
M3	144,448 ± 56,162	423,439 ± 93,059	85,245 ± 16,702
I1	140,858 ± 94,768	627,179 ± 137,246	176,392 ± 74,810
I3	39,783 ± 17,599	204,914 ± 49,099	61,945 ± 34,492
I4	68,678 ± 11,645	147,613 ± 44,903	45,723 ± 7,610
I5	37,027 ± 21,463	62,221 ± 13,380	54,634 ± 23,587
B1	198,893 ± 57,286	429,418 ± 43,503	202,694 ± 75,156
B3	184,112 ± 51,392	488,947 ± 86,189	161,864 ± 70,596
B4	183,470 ± 49,454	317,944 ± 79,980	274,012 ± 88,414
B6	222,515 ± 105,034	456,303 ± 87,734	273,466 ± 101,442
Mth	201,230 ± 84,794	395,530 ± 90,345	139,834 ± 31,750

Refers to Fig. 1 and Suppl. Figure 2

### GABA and baclofen treatments inhibit caspase activation in descending neurons after SCI

**Fig. 2:**
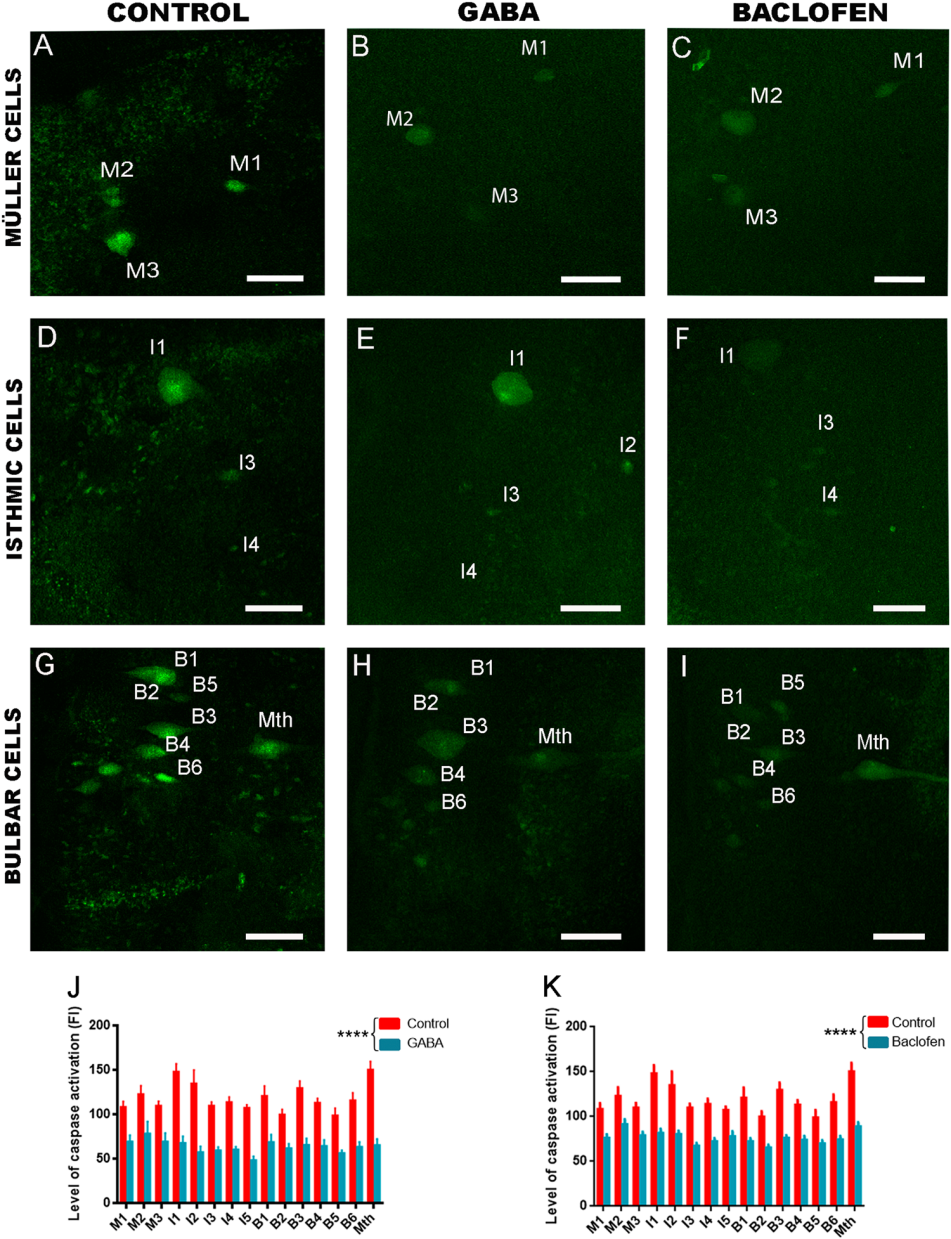
GABOB and baclofen treatments inhibit caspase activation in identifiable descending neurons. **a**, **d** and **g** Photomicrographs of whole-mounted brains showing identifiable descending neurons with intense FLICA labelling in control animals. **b**, **e** and **h** Photomicrographs of whole-mounted brains showing identifiable descending neurons with a reduction in FLICA labelling in GABA-treated animals. **c**, **f** and **i** Photomicrographs of whole-mounted brains showing identifiable descending neurons with a reduction in FLICA labelling in baclofen-treated animals. **j** Graph showing significant changes (asterisks) in the level of caspase activation (intensity of fluorescent FLICA labelling) after the GABA treatment. **k** Graph showing significant changes (asterisks) in the level of caspase activation (intensity of fluorescent FLICA labelling) after the baclofen treatment. Rostral is up in all photomicrographs. Scale bars: 100 µm

To test our hypothesis, we first analyzed the effect of GABA and baclofen (GABAB agonist) treatments in caspase activation in identifiable descending neurons after a complete SCI using FLICA labelling (Fig. 2a–i). As previously shown, in control lesioned animals, there is a statistically significant correlation between the intensity of FLICA labelling and the long-term survival and regenerative abilities of identifiable neurons (not shown;^18,50^). At 2 wpl, animals treated with GABA or baclofen during 4 days showed a significant inhibition of caspase activation (fluorescence intensity of FLICA labelling) in identifiable descending neurons as compared to control animals (GABA: Student’s *t*-test, *p* < 0.0001; baclofen: Student’s *t*-test, *p* < 0.0001; Fig. 2j, k). This suggests that GABA can inhibit apoptosis in descending neurons after SCI by activating GABAB receptors.

### GABOB and baclofen long-term treatments promote axonal regeneration in descending neurons after SCI

**Fig. 3:**
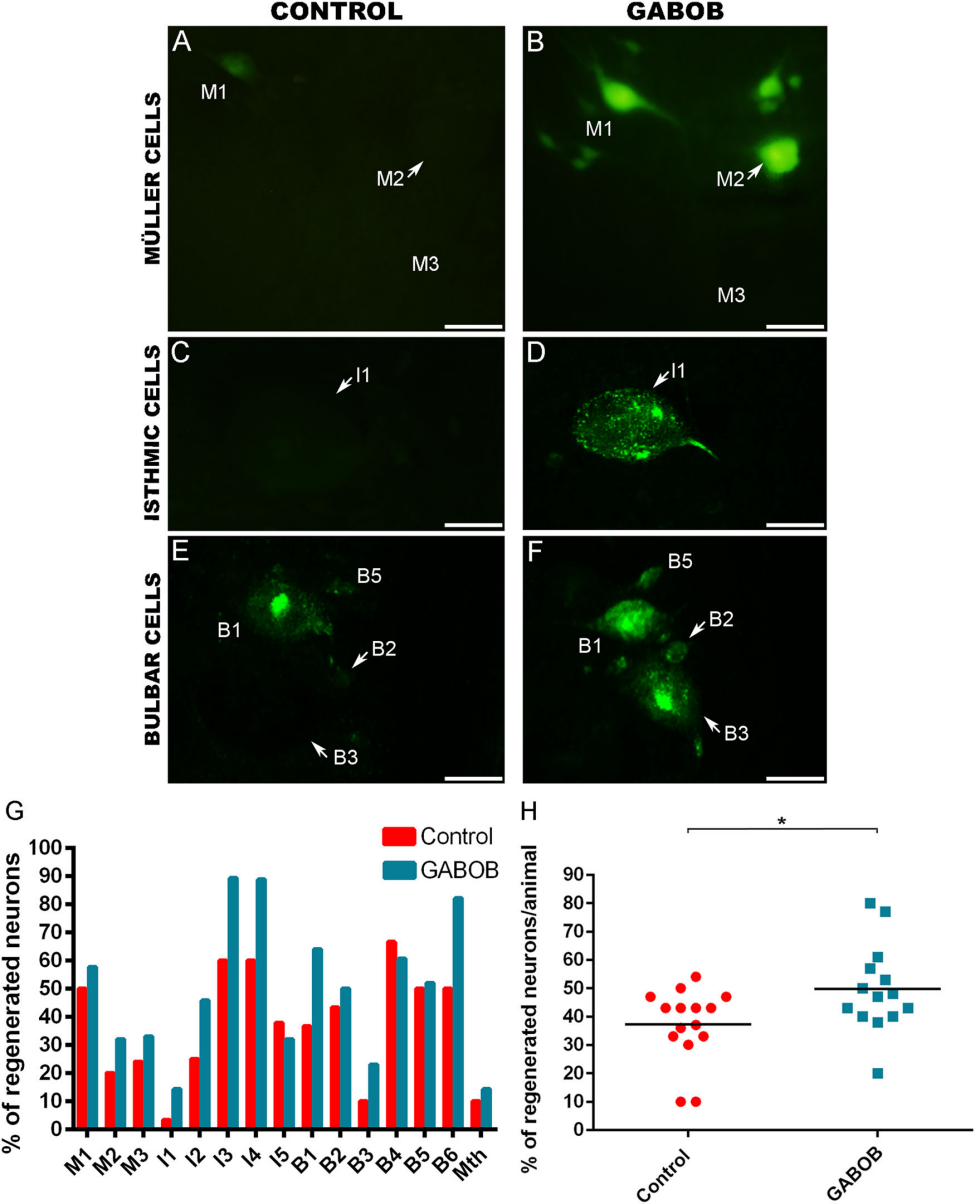
A long-term GABOB treatment promotes axonal regeneration of identifiable descending neurons. **a**, **c** and **e** Photomicrographs of whole-mounted brains showing different reticulospinal populations with regenerated identifiable neurons in control animals, as identified by retrograde labelling. **b**, **d** and **f** Photomicrographs of whole-mounted brains showing different reticulospinal populations with an increased number of labelled (regenerated) identifiable neurons in treated animals. **g** Graph showing the percentage of regenerated neurons (with respect to the total number of analyzed neurons) for each identifiable cell in control and GABOB-treated animals. **h** Graph showing significant changes (asterisks) in the percentage of regenerated neurons per animal after the GABOB treatment (control: 37.27 ± 3.33%; GABOB: 49.79 ± 4.16%). Arrows indicate descending neurons that regenerated in GABOB-treated animals but not in controls animals. Rostral is up in all photomicrographs. Scale bars: 50 µm

**Fig. 4:**
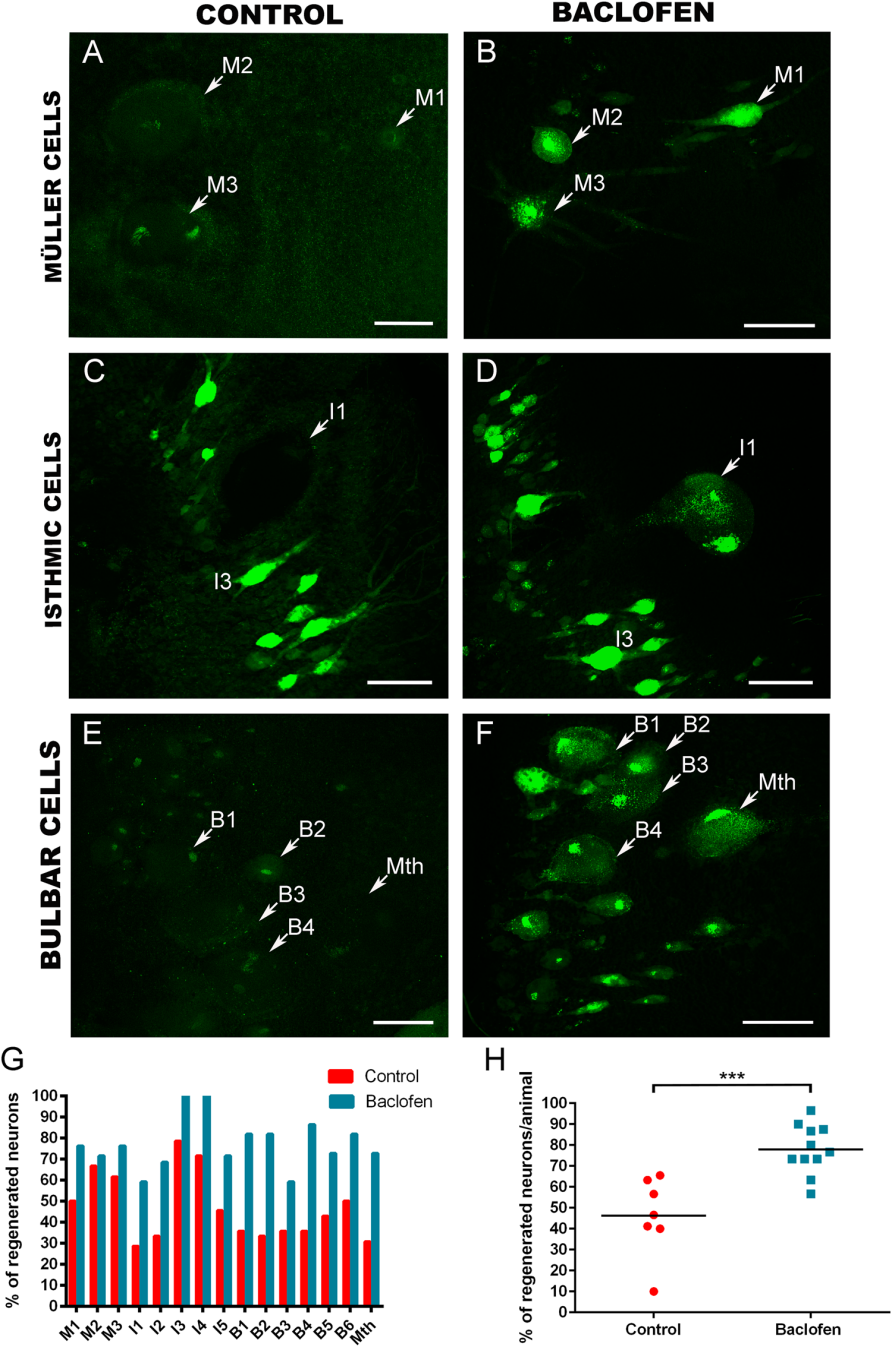
A long-term baclofen treatment promotes axonal regeneration of identifiable descending neurons. **a**, **c** and **e** Photomicrographs of whole-mounted brains showing different reticulospinal populations with regenerated identifiable neurons in control animals. **b**, **d** and **f** Photomicrographs of whole-mounted brains showing different reticulospinal populations with an increased number of labelled (regenerated) identifiable neurons in treated animals. **g** Graph showing the percentage of regenerated neurons (with respect to the total number of analyzed neurons) for each identifiable cell in control and baclofen-treated animals. **h** Graph showing significant changes (asterisks) in the percentage of regenerated neurons per animal after the baclofen treatment (control: 46.17 ± 7.15%; baclofen: 77.91 ± 3.56%). Arrows indicate descending neurons that regenerated in baclofen-treated animals but not in controls animals. Rostral is up in all photomicrographs. Scale bars: 100 µm

Then, we studied the long-term effect of increasing GABAergic signalling in axonal regeneration after a complete SCI. Retrograde neuronal tract-tracing with Neurobiotin showed that a treatment with either GABOB (Fig. 3) or baclofen (Fig. 4) during 12 weeks post-lesion significantly promoted axonal regeneration of identifiable descending neurons after a complete SCI as compared to control animals (GABOB: Student’s *t*-test, *p* = 0.0129 (Fig. 3h); baclofen: Mann–Whitney *U* test, *p* = 0.0004 (Fig. 4h)). The baclofen used in these experiments (from Carbosynth) was also tested to confirm that it had the same effect in the activation of caspases as the baclofen acquired from Molekula. This baclofen also inhibited caspase activation significantly in identifiable descending neurons as compared to control animals in a different set of experiments (Mann–Whitney *U* test, *p* < 0.0001; not shown). This shows that an increase in GABAergic signalling through GABAB receptors promotes axonal regeneration after a complete SCI.

### Endogenous GABA signalling through GABAB receptors promotes axonal regeneration after SCI

**Fig. 5:**
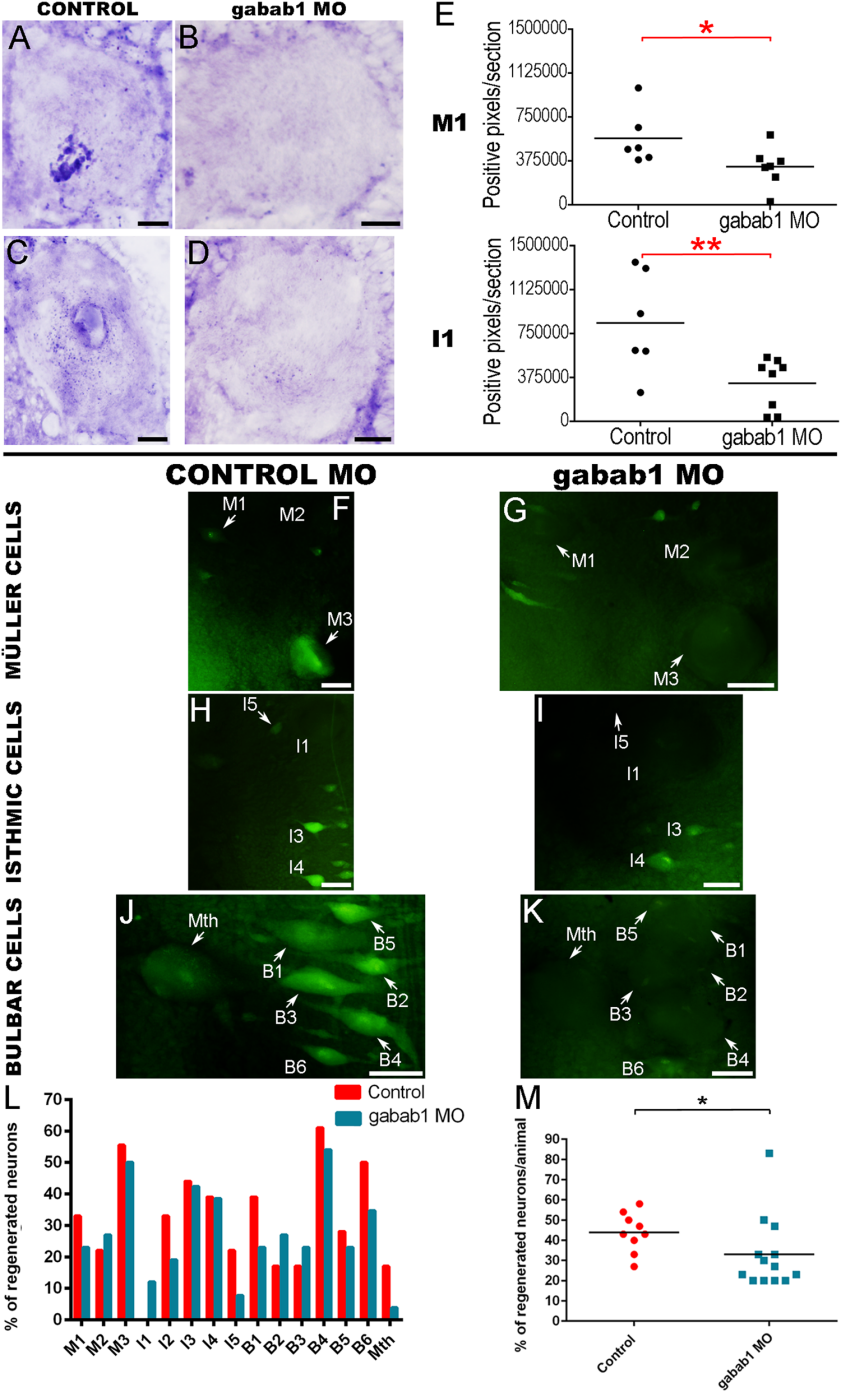
Gabab1 morpholino treatments inhibit axonal regeneration of identifiable descending neurons. **a**, **c** Photomicrographs of transverse sections of M1 (**a**) and I1 (**c**) neurons showing the expression of the gabab1 transcript in control animals. **b**, **d** Photomicrographs of transverse sections of M1 (**b**) and I1 (**d**) neurons showing the decreased expression of gabab1 transcript in gabab1 morpholino-treated animals. **e** Graphs showing significant changes (asterisks) in the number of gabab1 positive pixels per section in the soma of M1 and I1 neurons after the gabab1 morpholino treatment. **f**, **h** and **j** Photomicrographs of whole-mounted brains showing different reticulospinal populations with regenerated identifiable neurons in animals treated with the control morpholino. **g**, **i**, **k**: Photomicrographs of whole-mounted brains showing different reticulospinal populations with fewer regenerated identifiable neurons in animals treated with the active gabab1 morpholino. **l** Graph showing the percentage of regenerated neurons (respect to the total number of analyzed neurons) for each identifiable cell in control and active gabab1 morpholino-treated animals. **m** Graph showing significant changes (asterisk) in the percentage of regenerated neurons per animal after the morpholino treatment (control mismatch morpholino: 43.89 ± 3.26%; gabab1 active morpholino: 33 ± 5%). Arrows indicate descending neurons that regenerated in gabab1 morpholino-treated animals but not in controls animals treated with the mismatch control morpholino. Rostral is up in photomicrographs (**f**) to (**k**). Scale bars: black, 20 µm; white, 50 µm

To test whether endogenous GABA also promotes regeneration by activating GABAB receptors, we decided to use morpholinos to knockdown the expression of the gabab1 subunit in descending neurons after a complete SCI (Fig. 5). First, we used in situ hybridization to confirm that the active gabab1 morpholino is able to knockdown the expression of the gabab1 mRNA in identifiable neurons of 2 wpl animals (Fig. 5a–e). As an example, we analyzed the M1 (Fig. 5a, b) and the I1 neurons (Fig. 5c, d). The active gabab1 morpholino was able to significantly knockdown the expression of the gabab1 mRNA in identifiable descending neurons as compared to the gabab1 mismatch control morpholino (M1: Mann–Whitney *U* test, *p* = 0.0111; I1: Mann–Whitney *U* test, *p* = 0.0057; Fig. 5e). Then, neuronal tract-tracing showed that the treatment with the active gabab1 morpholino significantly inhibited axonal regeneration of identifiable descending neurons 10 weeks after a complete SCI as compared to the animals treated with the gabab1 mismatch control morpholino (Mann–Whitney *U* test, *p* = 0.0133; Fig. 5f–m). This confirms that, in lampreys, endogenous GABA promotes axonal regeneration of descending neurons after a complete SCI by activating GABAB receptors.

## Discussion

Here, we have provided gain and loss of function data, using pharmacological and genetic treatments, showing that endogenous GABA signalling through GABAB receptors promotes neuronal survival and axonal regeneration of identifiable descending neurons of lampreys after a complete SCI.

The analysis of the changes of expression of the gabab1 subunit in response to a complete SCI revealed a significant increase in the expression of this subunit in some identifiable descending neurons (with other neurons showing a similar trend). As stated in the introduction, massive glutamate release and the subsequent activation of glutamate receptors lead to an increase in Ca^2+^ influx into cells, which causes excitotoxicity and neuronal death after SCI^21,22,24,51^; see^52^. GABAB receptors can cause the inactivation of voltage-dependent Ca^2+^ channels (see^32^). Therefore, this increase in the expression of GABAB receptors could compensate for the influx of Ca^2+^ into axotomized descending neurons caused by massive glutamate release. The acute increase in the expression of the gabab1 subunit in descending neurons and the massive release of GABA after SCI^37^ appears as one of the mechanisms favouring neuronal survival and axonal regeneration after SCI in lampreys. As far as we are aware, no study has analyzed the expression of gabab subunits after SCI in mammals. Only a few mammalian studies have looked at changes in the expression of this receptor following other types of nervous system injuries (sciatic nerve ligation:^53^; traumatic brain injury:^54^; ulnar nerve transection:^55^; cerebral ischaemia:^56^). In contrast to the present results in lampreys, these studies showed that the expression of GABAB receptors decreases after the injury in different regions of the brain^54–56^. This could be a key difference between regenerating and non-regenerating animals, since axons of the later do not show good regenerative abilities after CNS injuries. Interestingly, and in agreement with the results in lampreys, Huang and colleagues^56^ reported that an elevation in the protein expression of GABAB receptors in the cerebral cortex promotes neuroprotection after ischaemic damage.

There is some controversy on the topic of whether descending neurons of the brain of mammals die after SCI. Some studies have shown the death of brain neurons after SCI^57–63^. On the other hand, two recent reports did not find evidence of the death of corticospinal neurons after SCI^64,65^, and suggested that these neurons only suffer atrophy but do not die^65^. In any case, the death or atrophy of descending neurons of mammals appears to involve apoptotic mechanisms as shown by the appearance of TUNEL labelling and activated caspase-3 immunoreactivity in these neurons after the injury at spinal levels^60–62^. Recent work in lampreys has also shown that identifiable descending neurons known to be “bad regenerators” are actually “poor survivors” after a complete SCI^8,12,14,50^. These neurons enter in a process of slow and delayed death after SCI^8,12–18^ that is initiated by caspase activation in the injured axon at spinal levels^17,18^. The death of these neurons also occurs through apoptotic mechanisms as shown by the appearance of activated caspases^15–18^, TUNEL labelling^14,15^ and Fluoro-Jade® C labelling^12,18^. Recent results have shown that there is a significant correlation between the intensity of caspase activation 2 wpl and the long-term regenerative^18^ and survival^50^ abilities of identifiable descending neurons of lampreys after SCI. Present results indicate that the activation of GABAB receptors by GABA/baclofen can inhibit caspase activation after SCI in identifiable descending neurons, which is a key step to preventing the development of apoptosis and promoting neuronal survival. Previous work in other models of CNS injury also showed that a baclofen treatment can inhibit caspase activation (model of kainic-acid-induced seizure in rats:^29^; models of ischaemic brain injury in rats:^27,31^; model of chemical hypoxia in retinal ganglion cells in rats:^66^). Our study shows that the activation of GABAB receptors can also prevent apoptosis after a traumatic SCI.

Of major importance is the fact that our results also support the role of GABA as a molecule that promotes true axonal regeneration of descending neurons through the site of a complete SCI. Behavioural analyses were not performed to establish a relationship between increased regeneration and improved functional recovery after the treatments. First, because in our case, control animals usually reach the highest level of recovery when using the Ayers test (see ref.^39^) and also because the treated animals were in the drugs until the day of analysis. Experiments using a gabab1 morpholino demonstrated that endogenous GABA acts as a pro-regenerative factor after SCI by activating GABAB receptors. The morpholino experiments suggest that GABA might promote regeneration by activating GABAB receptors expressed in the axotomized descending neurons. But, we cannot rule out the possibility that GABA could also promote the regeneration of descending neurons indirectly by inhibiting other cells expressing GABAB receptors, like intrinsic spinal cord neurons^39,42^ that could have also taken the morpholino in our experiments. Our data agree with previous in vitro or developmental studies regarding the role of GABA and GABAB receptors in neurite outgrowth^67^. López-Bendito and coworkers^67^ showed that the GABAB antagonist CGP52432 decreases the length of the leading process in migrating inhibitory neurons in brain slice cultures of mice. Also, both GABA and baclofen stimulate retinal ganglion neurite outgrowth in *Xenopus* cultures, and the GABAB antagonist CGP54262 shortened the developing optic projection in vivo^68^. But, as far as we are aware, our results are the first in vivo demonstration showing that GABA promotes axonal regrowth after a CNS injury by activating GABAB receptors. Present and previous^37^ results indicate that the GABAergic system of lampreys responds successfully to a SCI to limit retrograde degeneration and promote the regeneration of descending pathways.

## Conclusion

We have revealed a major role of GABA and GABAB receptors in promoting the survival and regeneration of individually identifiable descending neurons of lampreys following a complete SCI. Now, it would be of interest to decipher the underlying mechanisms behind the neuroprotective and pro-regenerative effect of GABA. Based on previous results in lampreys showing a negative effect of Ca^2+^ in neurite outgrowth^35,36^, a decrease in Ca^2+^ levels due to the activation of GABAB receptors could be one of the key events in the inhibition of apoptosis and activation of axonal regeneration by GABA. In future studies, it might be also interesting to analyze changes in gene expression elicited by GABA signalling and the activation of GABAB receptors to reveal new pathways involved in axonal regeneration and neuronal survival in lampreys.

The present results provide further support for the idea suggesting that the lesioned spinal cord is a “new spinal cord”^69^ and the importance of understanding the changes that occur after SCI in different neurotransmitter systems in the brain and in the spinal cord above and below the site of injury. This study adds to previous work revealing anatomical^37–39,70,71^ and physiological^40,72,73^ changes in different neurotransmitter systems above and below the lesion in recovered lampreys and highlights the importance of understanding these changes before applying neuropharmacological interventions in SCI patients. Specially, when drugs affecting neurotransmission might not only modulate locomotor circuits, but also affect the process of neuronal regeneration and recovery (e.g. serotonin inhibitors/toxins: see refs.^72,74^; GABOB/baclofen: present results).

Our results provide a strong basis to translate this knowledge to mammalian models of SCI for the development of new therapies for patients with SCI. A recent large observational cohort study has found that the early administration of gabapentinoids (which are administered as anticonvulsants for SCI patients) improves motor recovery following SCI^75^. Interestingly, baclofen is also already in use in the clinic, even for the treatment of SCI patients with spasticity^76^ or neuropathic pain^77^, which could facilitate the clinical translation of similar results in pre-clinical models of SCI.

## Electronic supplementary material

Supplementary Figure 1

Supplementary Figure 2

Supplementary Figure 3

Supplementary figure legends
