# Supplementary figures and images for: GABA promotes survival and axonal regeneration in identifiable descending neurons after spinal cord injury in larval lampreys
Source: Cell Death Dis. 2018 Jun 28;9(6):663. doi: 10.1038/s41419-018-0704-9 (PMC6021415; doi:10.1038/s41419-018-0704-9)

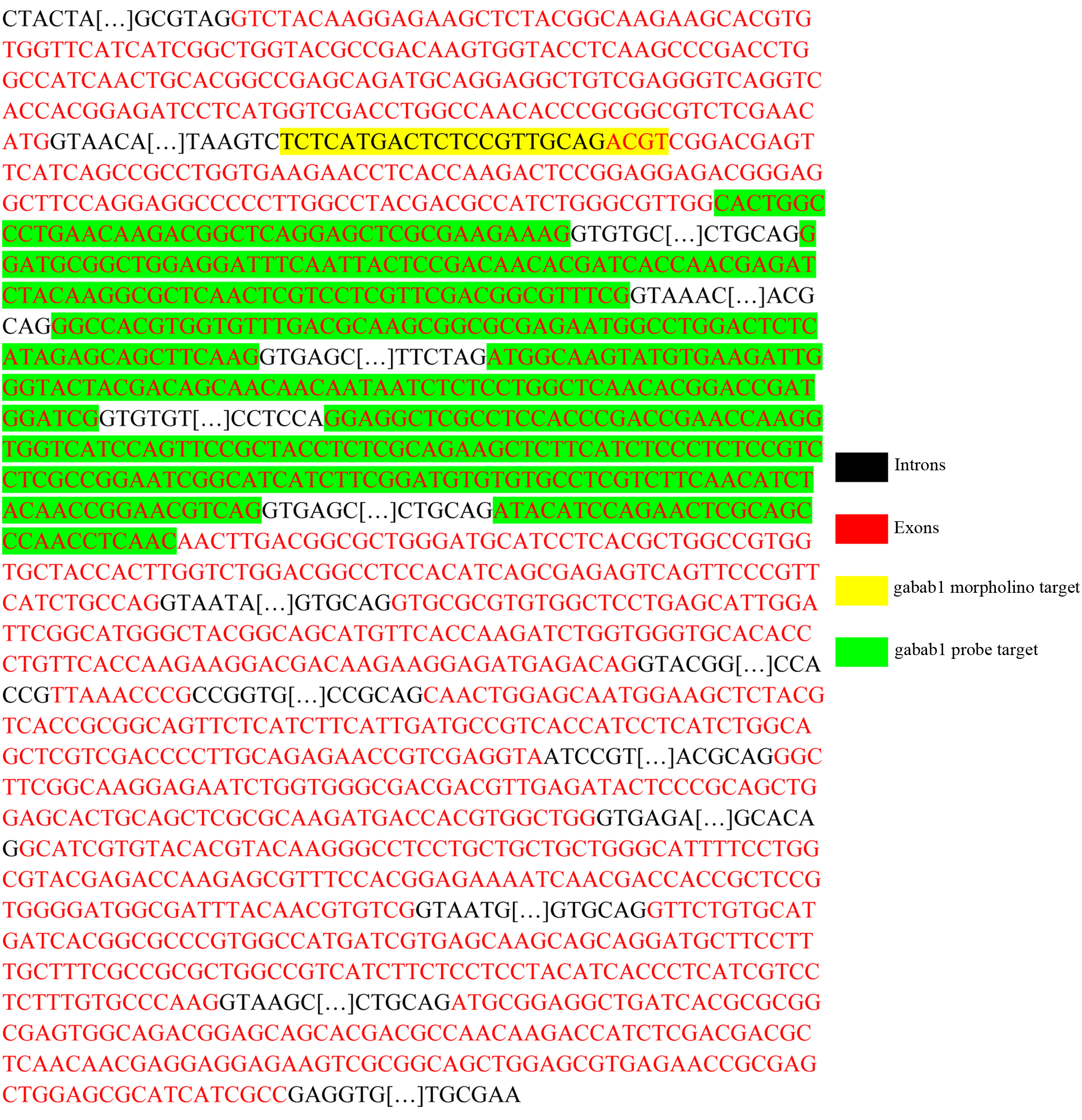

Supplement: Supplementary file 1 — Supplementary Figure 1 [file 41419_2018_704_MOESM1_ESM.tif]

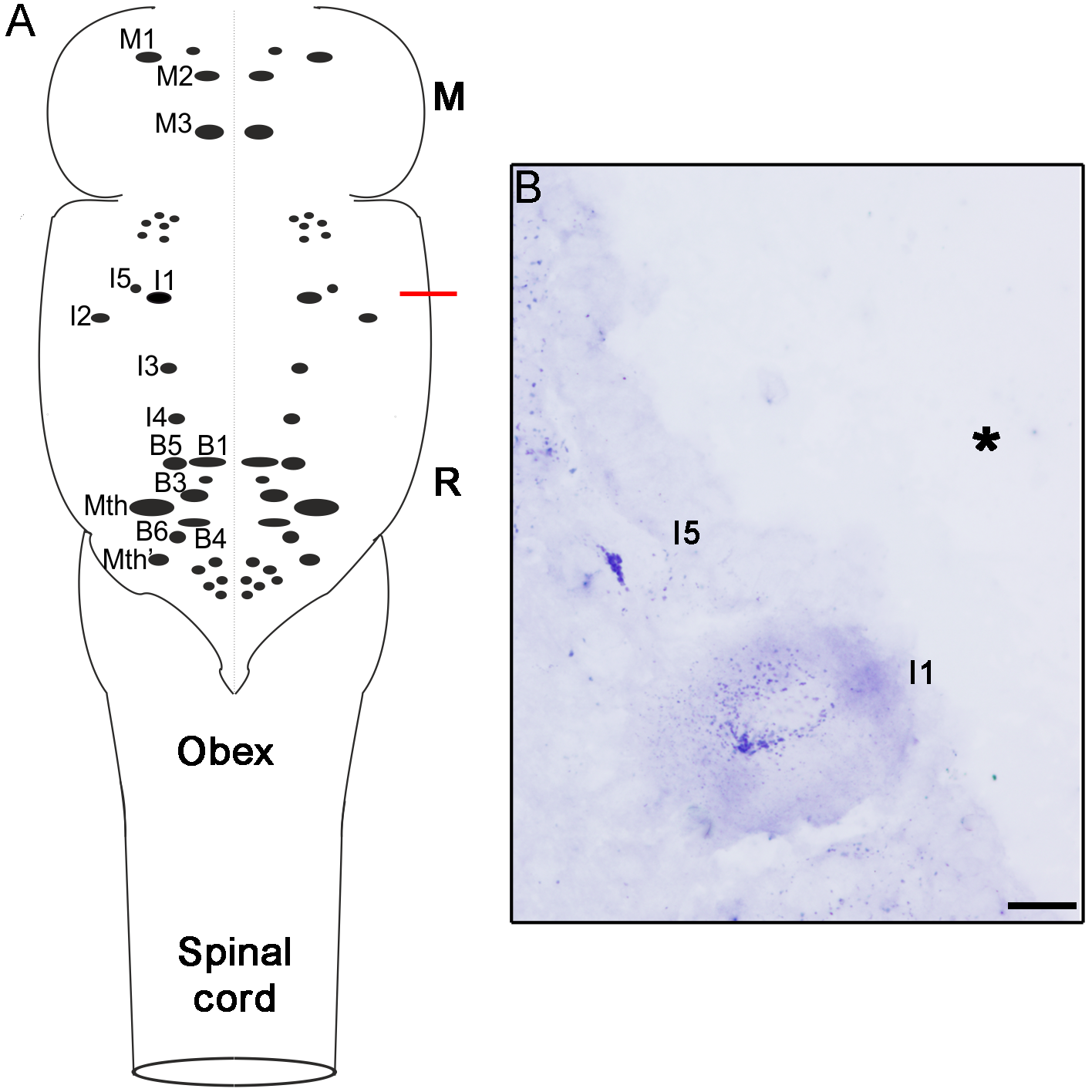

Supplement: Supplementary file 2 — Supplementary Figure 2 [file 41419_2018_704_MOESM2_ESM.tif]

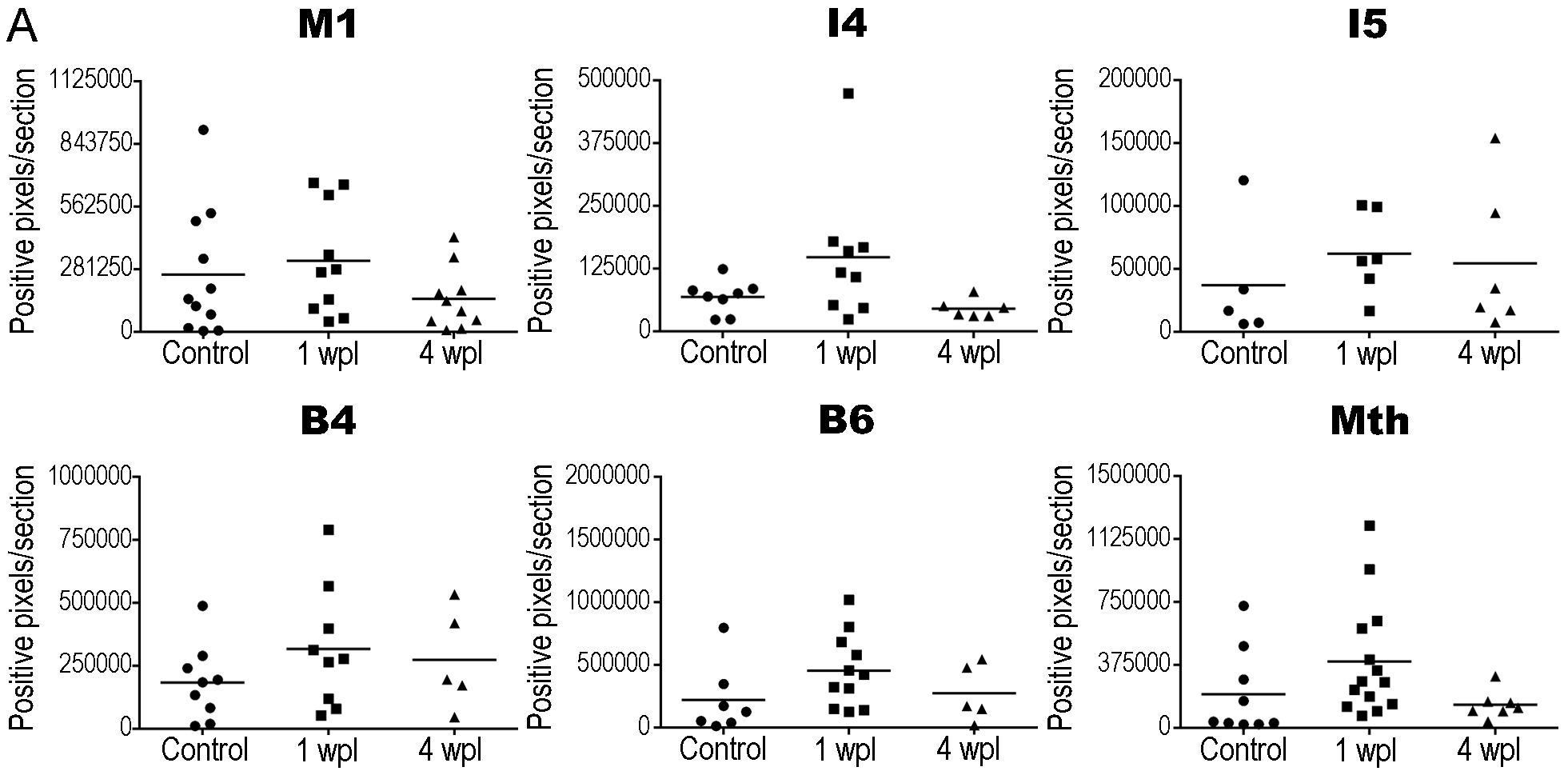

Supplement: Supplementary file 3 — Supplementary Figure 3 [file 41419_2018_704_MOESM3_ESM.tif]
