# Supplementary material for: GABA promotes survival and axonal regeneration in identifiable descending neurons after spinal cord injury in larval lampreys
Source: Cell Death Dis. 2018 Jun 28;9(6):663. doi: 10.1038/s41419-018-0704-9 (PMC6021415; doi:10.1038/s41419-018-0704-9)
Supplement: Supplementary file 4 — Supplementary figure legends [file 41419_2018_704_MOESM4_ESM.docx]

**Supplementary Figure 1.** Partial sequence of the sea lamprey gabab1 subunit gene, with exons in red and introns in black. Target sequence of the gabab1 *in situ* hybridization probe is highlighted in green. The target sequence of the gabab1 morpholino is highlighted in yellow (boundary of the second intron and second exon of the partial gabab1 sequence).

**Supplementary Figure 2. A:** Schematic drawing of a dorsal view of the sea lamprey brainstem showing the location of identifiable descending neurons (modified from [51]). **B:** Photomicrograph of a transverse section of the larval sea lamprey brain showing the expression of gabab1 subunit in identifiable descending neurons (I1 and I5) of the isthmic region of the rhombencephalon. The red line in A indicates the level of the transverse section in B. The asterisk indicates the ventricle. Rostral is to the top in A and dorsal is to the top in B. Abbreviations: M, mesencephalon; R, rhombencephalon. Scale bar: 20 µm.

**Supplementary Figure 3.** Graphs showing non-significant changes in the number of gabab1 positive pixels per section of the soma of identifiable descending neurons. The mean ± S.E.M. values are provided in table 2.
